# The PPCD1 Mouse: Characterization of a Mouse Model for Posterior Polymorphous Corneal Dystrophy and Identification of a Candidate Gene

**DOI:** 10.1371/journal.pone.0012213

**Published:** 2010-08-16

**Authors:** Anna L. Shen, Kathleen A. O'Leary, Richard R. Dubielzig, Norman Drinkwater, Christopher J. Murphy, Charles B. Kasper, Christopher A. Bradfield

**Affiliations:** 1 McArdle Laboratory for Cancer Research, School of Medicine and Public Health, University of Wisconsin-Madison, Madison, Wisconsin, United States of America; 2 Department of Ophthalmology and Visual Sciences, School of Medicine and Public Health, University of Wisconsin-Madison, Madison, Wisconsin, United States of America; 3 Eye Research Institute, School of Medicine and Public Health, University of Wisconsin-Madison, Madison, Wisconsin, United States of America; 4 Department of Surgical Science, School of Veterinary Medicine, University of Wisconsin-Madison, Madison, Wisconsin, United States of America; 5 Department of Pathobiological Sciences, School of Veterinary Medicine, University of Wisconsin-Madison, Madison, Wisconsin, United States of America; Johns Hopkins School of Medicine, United States of America

## Abstract

The PPCD1 mouse, a spontaneous mutant that arose in our mouse colony, is characterized by an enlarged anterior chamber resulting from metaplasia of the corneal endothelium and blockage of the iridocorneal angle by epithelialized corneal endothelial cells. The presence of stratified multilayered corneal endothelial cells with abnormal patterns of cytokeratin expression are remarkably similar to those observed in human posterior polymorphous corneal dystrophy (PPCD) and the sporadic condition, iridocorneal endothelial syndrome. Affected eyes exhibit epithelialized corneal endothelial cells, with inappropriate cytokeratin expression and proliferation over the iridocorneal angle and posterior cornea. We have termed this the “mouse PPCD1” phenotype and mapped the mouse locus for this phenotype, designated “*Ppcd1*”, to a 6.1 Mbp interval on Chromosome 2, which is syntenic to the human Chromosome 20 *PPCD1* interval. Inheritance of the mouse PPCD1 phenotype is autosomal dominant, with complete penetrance on the sensitive DBA/2J background and decreased penetrance on the C57BL/6J background. Comparative genome hybridization has identified a hemizygous 78 Kbp duplication in the mapped interval. The endpoints of the duplication are located in positions that disrupt the genes *Csrp2bp* and *6330439K17Rik* and lead to duplication of the pseudogene *LOC100043552*. Quantitative reverse transcriptase-PCR indicates that expression levels of *Csrp2bp* and *6330439K17Rik* are decreased in eyes of PPCD1 mice. Based on the observations of decreased gene expression levels, association with *ZEB1*-related pathways, and the report of corneal opacities in *Csrp2bp^tm1a(KOMP)Wtsi^* heterozygotes and embryonic lethality in nulls, we postulate that duplication of the 78 Kbp segment leading to haploinsufficiency of *Csrp2bp* is responsible for the mouse PPCD1 phenotype. Similarly, *CSRP2BP* haploinsufficiency may lead to human PPCD.

## Introduction

The inherited human corneal endothelial dystrophies, posterior polymorphous corneal dystrophy (PPCD), congenital hereditary endothelial dystrophy (CHED), and Fuchs endothelial dystrophy (FECD), are characterized by abnormal development, dysfunction and/or proliferation of the corneal endothelium [1], [2], [3]. These corneal dystrophies and the sporadic disorder, iridocorneal endothelial syndrome (ICE), exhibit overlapping clinical and pathological features, including ultrastructural changes and abnormal patterns of cytokeratin expression consistent with epithelialization[4], [5], [6]. Human PPCD is characterized by the presence of abnormal corneal endothelial cells which display epithelial features including microvilli and inappropriate cytokeratin expression[2], [6], [7] Clinical outcomes vary from minimal visual impairment to an aggressive course, with development of retrocorneal membranes and corneal opacification requiring keratoplasty [8], [9], [10]. Glaucoma has been reported at frequencies ranging from 10%–40% in different PPCD pedigrees [11]. ICE is also associated with increased intraocular pressure and development of glaucoma [12].

Human PPCD, like many eye disorders, exhibits genetic heterogeneity and has been linked to three chromosomal loci, 10p11, 20p11.2, and 1p34.3-p32. The transcription factor *ZEB1* gene, at 10p11, is the best characterized PPCD gene (*PPCD3*, MIM #609141) [13], [14], [15], [16]. Recently, missense mutations in *ZEB1* in association with a locus on chromosome 9 have also been linked to FECD [17]. In addition, PPCD in a number of families has been localized to an interval on chromosome 20p11.2 (*PPCD1*, MIM #122000) [11], [18], [19] that overlaps the region linked to CHED1, the autosomal dominant form of CHED [19], [20]. The coding regions of the 26 human *PPCD1* candidate genes located in the region common to three *PPCD1* pedigrees, plus 9 adjacent genes linked to CHED1, have been sequenced, but no causative mutations have been found [21], [22], [23]. Finally, mutations in the *COL8A2* gene, located on chromosome 1 and encoding the alpha-2 chain of type VIII collagen, have been associated with both PPCD (*PPCD2*, MIM #609140) and FECD [24], [25].

In the course of gene targeting studies designed to disrupt the murine *Cypor* locus [26], we observed mice with enlarged eyes in progeny derived from one of the targeted embryonal stem (ES) cell clones. This phenotype, which we termed mouse PPCD1, segregated independently from the targeted *Cypor* gene and appeared to be the result of a spontaneous mutation at an unknown location within the genome of the ES cells. PPCD1 mice exhibit an enlarged anterior chamber as a consequence of epithelialization of the corneal endothelium and proliferation of these epithelialized cells into the iridocorneal angle. These characteristics closely resemble that observed in the human PPCD and ICE. The possibility that the PPCD1 mouse could serve as a model for human PPCD or ICE led us to characterize the murine phenotype and identify its genetic basis.

## Results

### Origin of the PPCD1 mouse

**Figure 1:**
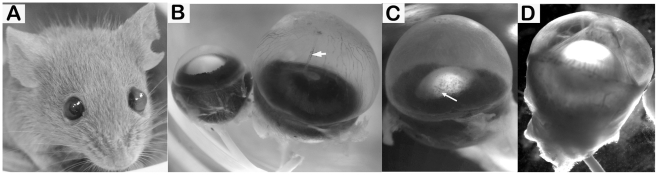
Phenotypic appearance of mouse PPCD1. Enucleated eyes were fixed in 10% formalin in phosphate-buffered saline and photographed. A. Appearance of PPCD1 mouse, showing the enlarged anterior chamber. B. Comparison of normal (left) and affected (right) eyes. Corneal neovascularization can be observed on the surface of the affected cornea. The arrow indicates an anterior synechia. Age, 3 months. C. Affected eye, showing corneal haze and cell growth across the surface of the pupil (arrow). Age, 3 months. D. Affected eye, showing lens subluxation, corneal haze, exudate in the anterior chamber, and anterior synechia. Age, 5 months.

Four founder males, all derived from the ES cell clone designated G1, gave rise to progeny exhibiting the phenotype of an enlarged anterior chamber that we designate as mouse PPCD1 (Fig. 1A). Both eyes are affected and males and females are equally affected. Animals derived from a separate ES clone (H11) from these same *Cypor* targeting experiments did not exhibit enlarged eyes. Initial ophthalmoscopic examinations to characterize the mouse PPCD1 phenotype were carried out on 3–5-month-old animals (49 PPCD1 and 30 normal littermates) on the mixed 129/B6 background. These exams revealed a deep anterior chamber and corneal abnormalities including corneal haze, neovascularization, ulcers and scarring. Posterior and anterior synechiae, lens subluxation, and phthisis were also observed. None of these phenotypes were observed in normal littermates. Genotyping established that the PPCD1 phenotype was independent of the targeted *Cypor* allele. Absence of the *neo* resistance cassette in PPCD1 animals was also confirmed (data not shown). All subsequent studies were carried out using PPCD1 animals wild-type at the *Cypor* locus.

### Anterior segment abnormalities of PPCD1 mice

Microscopic examination at low power of enucleated, formalin-fixed eyes from 3-month-old PPCD1 animals backcrossed to DBA/2J for 8 generations reveals an increase in the depth of the anterior chamber (Figure 1B). Measurements of fixed eyes from 3-month-old animals indicate that the increased depth of the anterior chamber results in a 41% increase in the overall length of affected eyes, from 3.34±0.13 mm (n = 10) in normal animals, to 4.70±0.35 mm (n = 10) in PPCD1 littermates (P<0.001). The overall diameter of the eye is also increased, by 19%, from 3.30±0.08 mm to 3.91±0.16 mm (P<0.001). Other findings shown in Figure 1 include corneal haze and neovascularization (observed in over 80% of affected eyes), corneal scarring, proliferation of cells over the surface of the lens, lens subluxation, synechiae, and phthisis. None of these abnormalities are observed in normal littermates.

**Figure 2:**
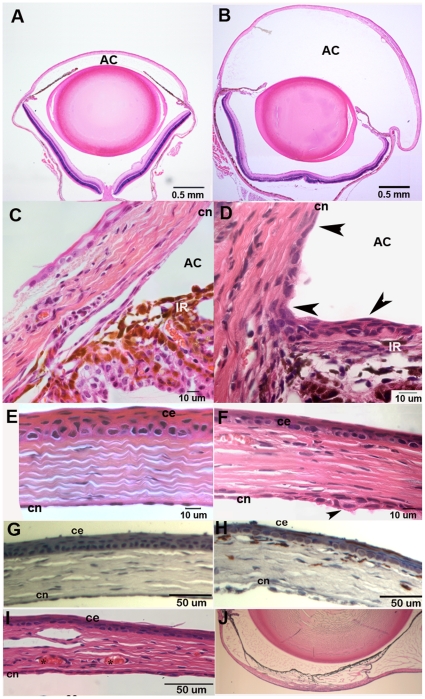
Histology of affected eyes. Eyes were fixed in 10% formalin in phosphate-buffered saline, processed, and stained with hematoxylin and eosin as described in Materials and Methods. Genotypes are indicated at the upper left. D2, DBA/2J; AC, anterior chamber; c, cornea; ce, corneal epithelium; cn, corneal endothelium; Ir, iris; s, synechia. A. Normal eye, age 2.5 months. B. PPCD1 eye, age 2.5 months. C. Normal iridocorneal angle, age 3 months. D. PPCD1 iridocorneal angle, age 3 months. Arrowheads indicate multilayered, stratified endothelial cells. E. `Normal cornea, age 3 months. F. PPCD1 cornea showing multilayered, stratified endothelial cells on the posterior surface of the central cornea (arrowhead), age 3 months. G. F4/80 immunohistochemistry of normal cornea, age P19. H. F4/80 immunohistochemistry of PPCD1 cornea, age P19. F4/80-positive cells stain reddish-brown. I. Corneal neovascularization (asterisks), age 3 months. J. Anterior synechia, age 5 months.

Histological examination of paraffin sections from PPCD1 eyes reveals distension of the anterior chamber (Figure 2B) and occlusion of the trabecular meshwork by multilayered, stratified endothelial cells (Figure 2D). These cells exhibit an epithelial morphology and are visible in the iridocorneal angle and on the posterior surface of the cornea and anterior surface of the iris (Figure 2D). Migration of these abnormal cells onto the central cornea is also observed (Figure 2F). In some affected eyes, thinning of the central cornea is observed. Corneas of PPCD1 animals, but not normal littermates, also display F4/80-immunoreactive cells, indicative of increased macrophage infiltration (Figure 2H). Other features associated with the mouse PPCD1 phenotype include corneal neovascularization (Figure 2I) and anterior synechias (Figure 2J).

**Figure 3:**
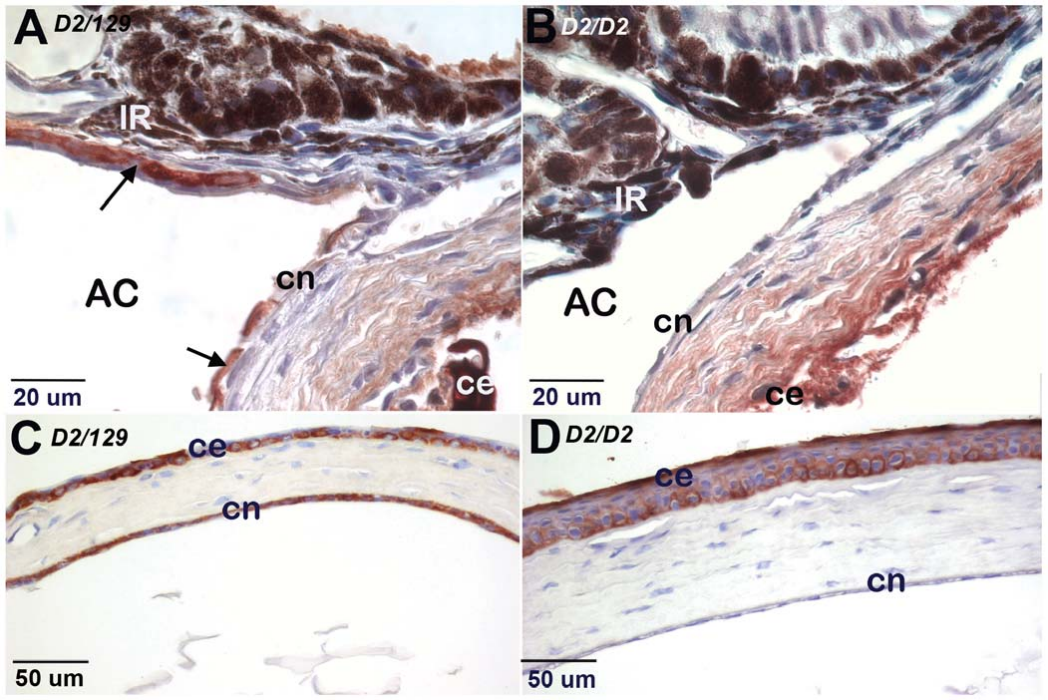
Cytokeratin AE1/AE3 immunohistochemistry of affected eyes. Positive cells are reddish-brown. Genotypes are indicated at the upper left. D2, DBA/2J; ce, corneal epithelium; cn, corneal endothelium; AC, anterior chamber; IR, iris. A. Iridocorneal angle of PPCD1 animal, age P19. Arrows indicate epithelialized endothelium. B. Iridocorneal angle of normal littermate, age P19, showing absence of immunoreactivity in the corneal endothelium and iridocorneal angle. C. Central cornea of 3-month old PPCD1 animal showing cytokeratin AE1/AE3 immunoreactivity of both corneal endothelium and epithelium. D. Central cornea of 3-month old normal littermate showing cytokeratin AE1/AE3 immunoreactivity of corneal epithelium only.

In PPCD1 animals, affected eyes exhibit inappropriate cytokeratin AE1/AE3 immunoreactivity in the corneal endothelium and iridocorneal angle, extending onto the anterior surface of the iris and posterior surface of the cornea (Figure 3A). Figure 3C shows cytokeratin AE1/AE3-positive cells extending across the posterior surface of the central cornea in a 3-month old PPCD1 animal. Eyes from normal littermates exhibit appropriate patterns of cytokeratin AE1/AE3 immunoreactivity, with positive staining of corneal epithelium but not corneal endothelium (Figure 3, B and D).

### Effect of genetic background on the PPCD1 phenotype

To assess the effects of genetic background on penetrance of the PPCD1 phenotype, PPCD1 mice on the DBA/2J background (N8 generation) were mated with either FVB/N or C57BL/6J (B6) mice. Frequencies of animals with enlarged eyes were 38% (n = 91, P<.05) and 44% (n = 44, P<0.2), respectively, for the PPCD1 x B6 F_1_ and PPCD1 x FVB/N F_1_ animals, consistent with autosomal dominant transmission and decreased penetrance on the B6 background. Further backcrossing to B6 resulted in loss of the PPCD1 phenotype after three generations of backcrossing. The size of the anterior chamber in affected F_1_ animals was comparable to that seen on the DBA/2J background.

### Development of the mouse PPCD1 phenotype

**Figure 4:**
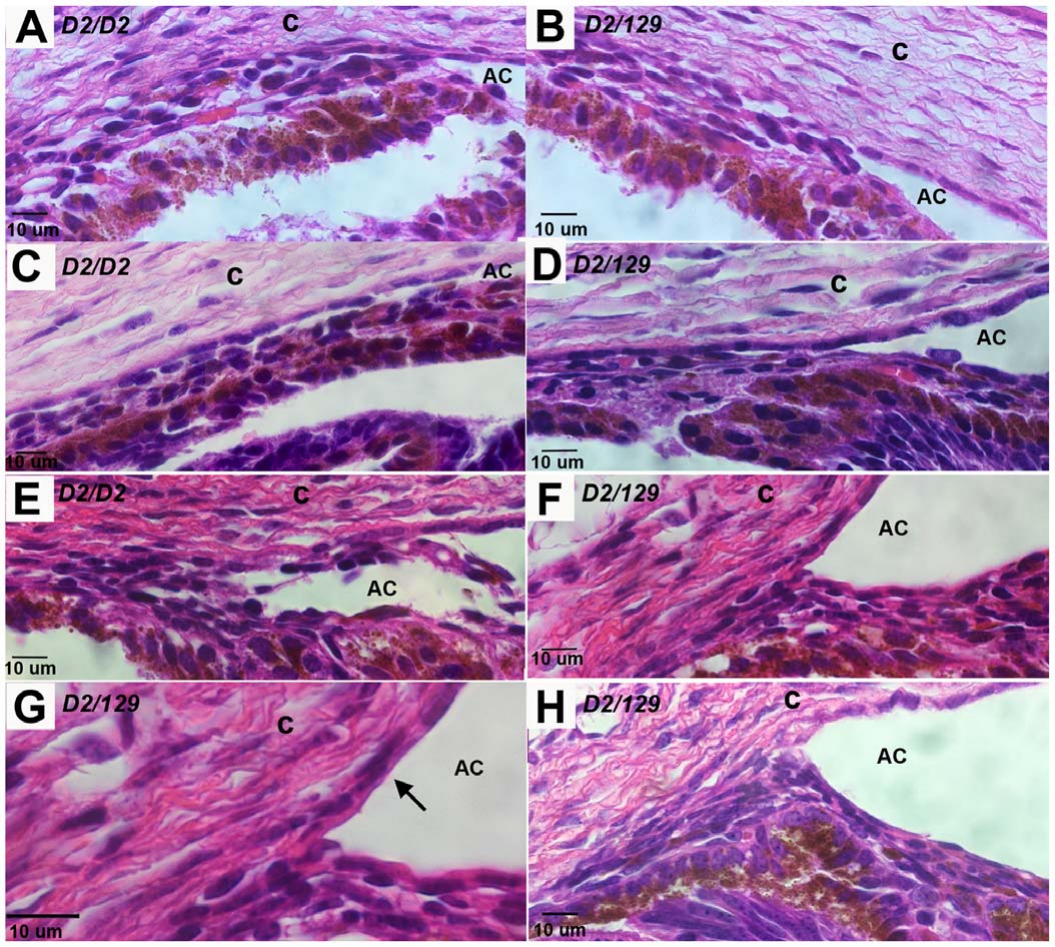
Development of the mouse PPCD1 phenotype. The iridocorneal angle from normal eyes (A, C, and E) is compared with that of PPCD1 littermates (B, D, F, and G). Panel H shows the PPCD1 iridocorneal angle at P5. Genotypes are indicated at the upper left. D2, DBA/2J; AC, anterior chamber; c, cornea. A. Normal, P0. B. PPCD1, P0. C. Normal, P2. D. PPCD1, P2. E. Normal, P3. F. PPCD1, P3. G. PPCD1, P3, enlarged to show layering of corneal endothelial cells (arrow). H. PPCD1, P5.

Histological examination of the developing iridocorneal angle immediately after birth and at postnatal day 2 (P2) reveals no apparent differences between wild-type and PPCD1 (Fig. 4, B and D). At P3, stratified and multilayered corneal endothelial cells are visible and cells in the iridocorneal angle appear to be more densely packed (Fig. 4, F and G). At P5, the iridocorneal angle is obviously occluded (Fig. 4H), with observable distension of the anterior chamber.

### Chromosomal localization of mouse PPCD1

**Figure 5:**
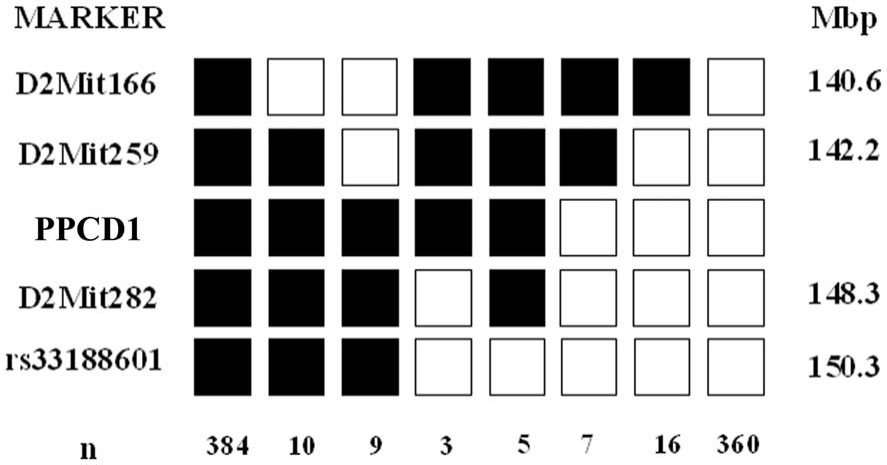
Chromosomal localization of *Ppcd1* locus. Shaded boxes indicate the129 (PPCD1) allele. Open boxes indicate the DBA/2J (normal eye) allele. SSLP markers analyzed are indicated on the left. Numbers of animals of each haplotype are indicated at the bottom.

In crosses between PPCD1 and DBA/2J mice (N5 through N8, n = 204), 52% of the offspring exhibited big eyes, consistent with autosomal dominant inheritance at a single locus, designated *Ppcd1*, and complete penetrance in the DBA/2J background. Linkage of the *Ppcd1* mutation to agouti was observed in the initial backcrossing experiments. This was confirmed by genotyping with the simple sequence length polymorphism (SSLP) markers *D2Mit22* and *D2Mit26*, located at 151.8 and 151.3 Mbp, respectively, on Chromosome 2, which indicated that 57/57 BE animals, but 0/28 normal animals, carried one 129 ES cell-derived allele at these loci. Further backcrossing (to N9) produced a nonagouti PPCD1 animal that was heterozygous for 129-derived markers from *D2Mit306*, at 135.1 Mbp, to *rs13470055*, at 150.44 Mbp. Linkage analysis of animals produced from backcrossing progeny of this animal to DBA/2J is presented in Figure 5 and places the *Ppcd1* mutation on Chromosome 2, between the markers *D2Mit259*, at 142.2 Mbp, and *D2Mit282*, at 148.3 Mbp.

In an effort to obtain homozygous null animals, PPCD1 heterozygous animals with enlarged eyes and carrying one 129-derived allele between the markers *D2Mit259 *and *D2Mit282* were intercrossed. Of a total of 149 progeny, no live pups homozygous for the 129-derived allele or displaying enlarged eyes have been found, suggesting that homozygosity of the *Ppcd1* mutation or a closely linked gene causes embryonic lethality. Genotyping of embryos at embryonic days 9.5 (n = 22) and 10.5 (n = 12) have also not yielded homozygous progeny.

### Comparative genome hybridization

**Figure 6:**
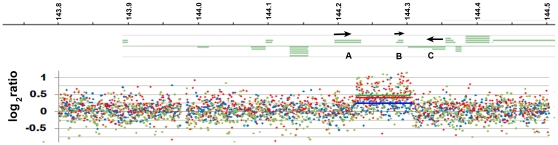
CGH Analysis. Log_2_ratio is shown in the lower panel and transcripts (green lines) in the top panel. Coordinates in Mbp are shown at the top. The log_2_ratio values are indicated by blue (PPCD1 versus DBA/2J), red (PPCD1 versus R1 ES cells), or green (G1 versus H11) dots. The solid lines indicate segments identified by the Nimblegen CGH-segMNT algorithm. Arrows indicate the direction of transcription. A, *Csrp2bp*; B, *LOC100043552*; C, *6330439K17Rik*.

To determine if a gross chromosomal rearrangement was responsible for the mouse PPCD1 phenotype, comparative genome hybridization was carried out on DNA from wild-type and PPCD1 animals using the Nimblegen 395 K Whole Genome 8 Array Tiling Set, which contains 385,000 50-75mer probes covering mouse Chromosome 2 from 124 Mbp to 181.9 Mbp at an average spacing of 656 bp. Three separate comparisons between PPCD1 and wild-type DNA each identified a single increased copy number segment on Chromosome 2 (Figure 6). Comparison of DNA from PPCD1 animals with wild-type littermates identified an 87,103 bp segment extending from 144,220,537 bp to 144,307,640 bp on Chromosome 2 and comprised of 122 probes. Comparison of DNA from PPCD1 animals with DNA from the 129/Sv-derived R1 ES cells used for gene targeting yielded essentially the same segment, extending from 144,225,647 bp to144,304,372 bp. Comparison of DNA from G1 and H11 ES cell clones, which gave rise to PPCD1 and normal progeny, respectively, also identified this segment, extending from 144,223,757 Mbp to 144,303,650 bp. The variability in the endpoints arises from inclusion or exclusion of 5 probes with log_2_ratios of approximately 0.2 at the endpoints of the segments by the CGH-segMNT algorithm. The 78 Kbp segment common to all three analyses, comprised of 108 probes, extends from 144,225,647 to 144,303,650 bp, with an uncertainty of 5 and 4 Kbp, for assignment of the proximal and distal breakpoints, respectively. The observed log_2_ratios of 0.24, 0.38, and 0.48 are consistent with the presence of a hemizygous duplication in the BE animals.

Two genes, *Csrp2bp* and *6330439K17Rik*, and one pseudogene, *LOC100043552*, are located in this segment. The predicted endpoints of the duplication are located between Exons 7 and 8 of *Csrp2bp* and between Exons 17 and 20 of *6330439K17Rik*, presumably disrupting both genes. *LOC100043552*, located in the center of the sequence, is presumably duplicated and not disrupted. No other Chromosome 2 copy number variations were detected on this chip. From our mapping data, it is highly unlikely that disruption of a gene outside the 6.1 Mbp interval is responsible for mousePPCD1; thus it is likely that one of these three genes is the *Ppcd1* gene.

**Figure 7:**
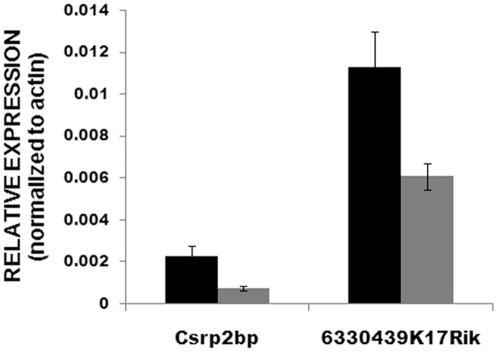
qPCR of *Csrp2bp* and *6330439K17Rik*. Expression levels were determined using IDT Prime Time^TM^ 5′- nuclease assays. Values are mean +/− SD. Black shows wild-type values and gray, PPCD1 values. P values are 0.01 and 0.02, for *Csrp2bp* and *6330439K17Rik*, respectively.

Microarray analysis (not shown) and qPCR of these three transcripts indicates that all three are transcribed and that expression of *Csrp2bp* and *6330439K17Rik* is decreased in PPCD1 eyes compared to wild-type at both 16 and 28 days. Figure 7 shows that expression levels of *Csrp2bp* and *6330439K17Rik* in PPCD1 eyes at 28 days are approximately half that of wild-type. Expression of *LOC100043552* is not significantly different between the two genotypes (not shown).

## Discussion

The mouse PPCD1 phenotype is characterized by abnormal growth of epithelialized corneal endothelial cells over the iridocorneal angle, posterior cornea, and iris and presents with early onset of an enlarged anterior chamber. The spectrum of ocular abnormalities associated with the mouse PPCD1 phenotype has not been previously observed in other mouse ocular mutants. The presence of corneal endothelial cells with epithelial features including microvilli and cytokeratin expression is a characteristic of the human autosomal dominant disorder PPCD, as well as ICE, a sporadic condition of unknown etiology [2], [6], [7].

Corneal endothelial cell metaplasia is the earliest observable mouse PPCD1 phenotype in this study, as shown by histology at postnatal day 3. We postulate that occlusion of the trabecular meshwork in the developing eye by epithelialized corneal endothelial cells leads to the visibly enlarged anterior chamber. Ocular enlargement, occlusion of the trabecular meshwork, and development of synechias are often associated with increased intraocular pressure and the question of whether PPCD1 mice develop glaucoma is currently under investigation.

The PPCD1 mouse arose spontaneously in the course of gene targeting experiments employing the R1 ES cell line. The mouse PPCD1 phenotype has not been reported in any of the large number of R1 - derived recombinant mouse lines that have been generated in our and other laboratories. Genetic analysis demonstrates that the mouse PPCD1 phenotype is unrelated to the presence of the *Cypor* null allele and the absence of the *neo* gene indicates that mouse PPCD1 is not due to a secondary insertion of the targeting vector at a nonhomologous site. Taken together, these observations suggest that the mouse PPCD1 phenotype is due to a spontaneous mutation in the G1 ES cell clone used in the *Cypor* targeting experiments.

As with human PPCD, transmission of the mouse PPCD1 phenotype is autosomal dominant. Murine null alleles of proposed human candidate genes, including *VSX1* [*27*] and *COL8A1* [28] null mice and the T27aT15 mouse[29], have not reproduced the corneal endothelial cell abnormalities seen in human PPCD. Recently, *Zeb1*-null embryos have been reported to exhibit ectopic expression of epithelial genes, abnormal corneal endothelial and keratocyte proliferation, corneal thickening, and corneolenticular and iridocorneal adhesions. Adult heterozygotes exhibit corneal thickening, increased keratocyte number, and iridocorneal and corneolenticular adhesions [30]. Like *Zeb1* heterozygotes, adult PPCD1 mice exhibit synechiae. However, the ocular phenotype of PPCD1 animals differs from that of *Zeb1*-haploinsufficient animals in that the phenotype is not morphologically apparent until after birth and proliferation of epithelialized corneal endothelial cells into the iridocorneal angle, with angle occlusion, is observed.

**Figure 8:**
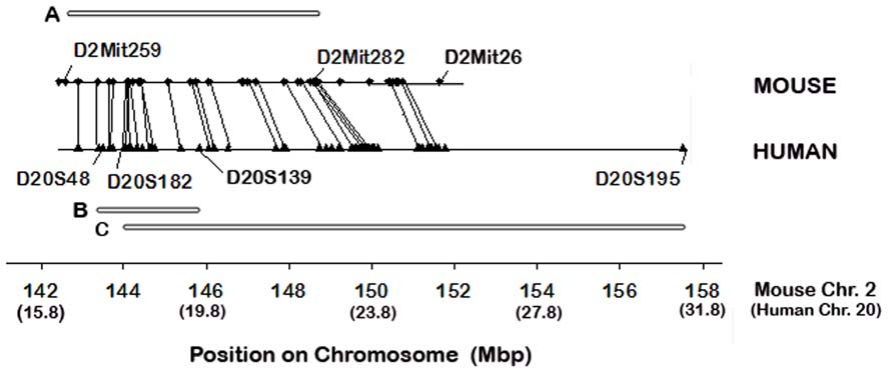
Comparison of mouse and human *PPCD1* loci. Mouse chromosome 2 is shown on the upper line and human chromosome 20 on the lower line. Chromosomal positions in Mbp are shown at the bottom, with the relative positions on human chromosome 2 shown in parentheses. Microsatellite markers used to define the mouse and human critical intervals are labelled. Bar A indicates the critical interval for mouse *Ppcd1* (see below) and bars B and C indicate the *PPCD1* critical intervals defined by Gwilliam *et al*. [18] and Yellore *et al*.[19], respectively. Mouse genes and markers are indicated by diamonds and human genes and markers are indicated by triangles. Lines connect orthologous genes.

Our mapping studies place the *Ppcd1* locus on mouse chromosome 2, between the markers *D2Mit259* and *D2Mit282*. This 6.1 Mbp region of Chromosome 2 is largely syntenic with the region on human chromosome 20 implicated in human PPCD (Fig. 8). Comparative genome hybridization reveals a 78 Kbp hemizygous duplication located between 144.2 and 144.3 Mbp, with some uncertainty in assignment of the endpoints. Regardless of the choice of endpoints, this duplication which has the potential to disrupt expression of 2 genes, *6330439K17Rik* and *Csrp2bp*, and produce two copies of the pseudogene *LOC100043552*. qPCR indicates that expression of *6330439K17Rik* and *Csrp2bp* are decreased in PPCD1 eyes compared to wild-type, while levels of *LOC100043552* are not different between the two genotypes.

*6330439K17Rik* is the mouse ortholog of human *C20ORF12*; both genes contain two ankyrin repeat-encoding regions (http://www.ncbi.nlm.nih.gov/cdd) and their function is unknown. Although *LOC100043552* is classified as a pseudogene based on the presence of multiple termination codons, it exhibits 81% DNA homology to its human ortholog, *ZNF133*, with conservation of the zinc finger motifs but not the KRAB domain. The *in vivo* function of *ZNF133* is unknown, but it has been shown to function as a transcriptional repressor *in vitro* [31].

*CSRP2BP*, also known as *ATAC2*, is a component of mammalian ATAC, a histone acetyltransferase complex shown to have essential functions in mammalian development [32], [33]. Another component of the ATAC complex is PCAF, a protein shown to be recruited by ZEB1 [34]. A *Csrp2bp*-null mouse, generated from 129-derived ES cells and maintained on the C57BL/6J background, has been reported to be embryonic-lethal at E9.5 [33]. No eye abnormalities in heterozygotes have been reported. However, a second, targeted knockout, *Csrp2bp^tm1a(KOMP)Wtsi^* , in which Exon 3 has been deleted, has been generated by the Wellcome Trust Sanger Institute and exhibits corneal opacities in heterozygotes and embryonic lethality in homozygotes (http://www.sanger.ac.uk/mouseportal/search/MGI:1917264. Based on the observations of decreased gene expression levels, association with *ZEB1* -related pathways, and the report of corneal opacities in heterozygotes and embryonic lethality in the null, we postulate that haploinsufficiency of *Csrp2bp* is responsible for the mouse PPCD1 phenotype.

The coding regions of human *C20ORF12, Csrp2bp and ZNF133* have been sequenced from DNA from the human PPCD1 pedigree described by Yellore *et al*. [19], [21], but no mutations segregating with PPCD in these families were identified. Mutations in coding regions are only one of a number of possible disease-causing genetic variations, as has been discussed by Aldave *et al.* [21].

Human developmental eye disorders are characterized by variable penetrance [35]; likewise genetic background influences the penetrance of the mouse PPCD1 phenotype. Penetrance of mouse PPCD1 is decreased in the C57BL/6J background compared to DBA/2J. DBA/2J mice are known to develop iris pigment dispersion and stromal atrophy leading to increased intraocular pressure and glaucoma. Although the anterior segment abnormalities seen in PPCD1 mice are distinct from those of DBA/2J, it is possible that common developmental pathways are affected.

In summary, the PPCD1 mouse is a model of human anterior segment dysgenesis with features of PPCD and ICE. Histological similarities, autosomal dominant transmission, and linkage to a region of chromosome 2 syntenic to the human *PPCD1* locus provide evidence that the PPCD1 mouse is a *bona fide* model of human PPCD. Epithelialization of the corneal endothelium and early onset distinguish the PPCD1 mouse from other mouse models of anterior segment dysgenesis. We have localized the *Ppcd1* gene to a 6.1 Mbp region of mouse chromosome 2 which is syntenic to a human PPCD locus. Comparative genome hybridization has identified a hemizygous duplication in this region which disrupts expression of two genes, *Csrp2bp* and *6330439K17Rik*. We propose that *Csrp2bp* is the *Ppcd1* gene and we are in the process of testing that hypothesis through development of corresponding mutant mouse alleles. The ability to manipulate expression of the mouse PPCD1 phenotype in different strains may provide important insights into how genes interact to influence ocular disease in mammalian systems.

## Methods

### Origin of the PPCD1 mouse

The PPCD1 mouse arose from gene targeting experiments designed to generate a null allele at the *Cypor* locus [26]. The R1 ES stem cell line employed in these experiments was derived from 129/Sv×129/Sv-CP embryos [36]. After injection of recombinant ES cells into B6 blastocysts, chimeras were crossed to the B6 strain to identify germ line transmission. Two separate ES cell clones, designated G1 and H11, gave rise to separate mouse lines that transmitted the targeting event at the *Cypor* locus. Four founder males derived from the G1 clone each gave rise to mice that displayed an enlarged anterior chamber. The H11 ES cell line gave rise to mice displaying normal ocular physiology. Animals derived from the G1 clone and exhibiting an enlarged anterior chamber, designated PPCD1, were maintained on a 129/B6 mixed background for 3 generations. To produce a congenic PPCD1 line on the DBA/2J background, PPCD1 animals wild-type at the *Cypor* locus were backcrossed to DBA/2J mice for 20 generations. PPCD1 animals were identified by the presence of a visibly enlarged anterior chamber (see Fig. 1A); all phenotype assignments were confirmed by examination of enucleated eyes under a dissecting microscope. To investigate the effect of genetic background, PPCD1 mice crossed onto the DBA/2J background for 8 generations were mated to either B6 or FVB/N mice and scored for the presence of an enlarged anterior chamber.

### Characterization of the mousePPCD1 phenotype

All procedures conformed to the principles embodied in the ARVO Statement for the Use of Animals in Ophthalmic and Vision Research (www.arvo.org). Procedures were also approved by the Animal Care and Use Committee, School of Medicine and Public Health, University of Wisconsin-Madison (Protocol Number M00578-0-11-07). Initial clinical characterization of the mouse PPCD1 phenotype was carried out on a group of 49 PPCD1 and 30 normal littermates on the mixed 129/B6 background (3–5 months old). Indirect ophthalmoscopy was performed using a Welch Allyn ophthalmic headset fitted with a 28 diopter Nikon individual lens. Mouse pupils were dilated with a solution containing 1.0% (w/v) tropicamide, 2.5% (w/v) phenylephrine, delivered using a blunted 30 gauge needle.

All subsequent phenotype scoring and characterization have been carried out by examination of formalin-fixed eyes under a dissecting microscope, using animals backcrossed to DBA/2J for at least 8 generations, Animals were sacrificed by CO_2_ inhalation and enucleated eyes were fixed overnight in 10% (v/v) formalin in phosphate-buffered saline, pH 7.2. Fixed eyes were rinsed with phosphate-buffered saline and scored under a dissecting microscope for the presence of ocular and anterior chamber enlargement, corneal neovascularization, corneal haze, synechiae, lens subluxation, corneal scarring, and phthisis. Animals exhibiting an enlarged anterior chamber were scored as PPCD1.

For histological characterization, eyes were formalin-fixed, dehydrated in graded ethanol, and processed for routine paraffin embedded light microscopy. Sections were stained with hematoxylin and eosin (H&E) or by immunohistochemistry. Primary antibodies used were anti-human cytokeratin [Clones AE1/AE3 (DAKO, Carpenteria CA)] or anti-mouse F4/80 (Serotec, Oxford UK). Primary antibody was omitted from negative controls. Following incubations with primary antibodies, sections were incubated with biotinylated secondary antibody and avidin-conjugated peroxidase using the Vectastain ABC kit and NovaRed substrate according to the manufacturer's instructions (Vector Laboratories, Burlingame CA).

### Genetic Mapping

Tail DNA was isolated using the Puregene reagent according to the manufacturer's instructions (Gentra, Minneapolis MN). G1 and H11 ES cell DNA was isolated from the expanded cell preparations used for microinjection into blastocysts. DNA from embryonal stem cells lines was isolated by Proteinase K and RNase digestion followed by phenol extraction and ethanol precipitation. Genotyping for the *Cypor* gene was carried out as described [26]. The Mouse Genome Informatics database (www.informatics.jax.org) was searched to identify SSLP and single nucleotide polymorphism (SNP) markers polymorphic for the 129X1/SvJ, C57BL/6J, and DBA/2J strains. The following SSLP markers were chosen for use in linkage analysis: *D2Mit 306, D2Mit259, D2Mit166, D2Mit282, D2Mit26, and D2Mit22*. Microsatellite markers were amplified using 1 µl DNA (∼20 ng), 0.2 µM each primer, 50 µM dNTPs, 1x polymerase chain reaction (PCR) buffer (Roche; 10 mM Tris–HCl, pH 8.3, 1.5 mM MgCl_2_, 50 mM KCl) and 0.5 U *Taq* polymerase in a final reaction volume of 20 µl. Reactions were incubated in an MJ PTC-200 thermal cycler at 94°C for 2 min and then for 40 cycles of 94°C for 30 sec, 55°C for 40 sec, 72°C for 60 sec and 72°C for 5 min. The products were separated by electrophoresis through a 4% MetaPhor agarose gel (Lonza). The SNP markers *rs33188601* and *rs13470055* were analyzed by PCR amplification of the region flanking the SNP followed by DNA sequencing. DNA sequencing was carried out using the BigDye protocol (Applied Biosystems) according to the manufacturer's directions; electrophoresis was carried out at the UW DNA Sequencing Facility. To confirm that these markers were indeed informative, each marker was tested on genomic DNA from DBA/2J, B6, and the G1 ES cells from which these animals were derived.

### Comparative genome hybridization

DNA from tails or ES cells was prepared as described above and analyzed by Nimblegen CGH Services. Tail DNA samples were prepared by pooling DNA from three wild-type or PPCD1 animals (littermates). Samples were hybridized to the MM8 WG CGH 2 of 8 (B4351-02-01) chip, which contains probes covering the following mouse DNA sequences: Chr2, 123,962,199 bp to 181,926,640 bp; Chr3, 3,000,451 bp to 159,871,498 bp; and Chr4, 3,006,937 bp to 108,173,152 bp. Data was analyzed by Nimblegen using their CGH-segMNT algorithm.

### Gene expression

Immediately after CO_2_ euthanasia, eyes were enucleated and placed in RNAlater (Qiagen). Total RNA was isolated using the Qiagen RNeasy Mini kit. First strand cDNA synthesis was carried with random hexamer primers and Superscript reverse transcriptase according to the manufacturer's directions. 5′-nuclease primer pairs and probes were designed and synthesized by Integrated DNA Technologies. For *Csrp2bp*, the primer pair used was 5′- ACATCCCAACCATCAACTCC -3′ and 5′-TCACATCAGGAACCATGAAGC-3′, with the labelled probe 5′-CAGACACTCAGACAGGTCAATGCCA-3′, which amplified a sequence extending from Exon 9 to Exon 10. For *6330429K17Rik*, the primer pair used was 5′-TGCCAC TGTCCATGAAGATG-3′ and 5′-GTCATATAGCCCGTTCCCAC-3′, with the labeled probe 5′-AAGAGCCTGTACTTCAACAAGTCGGTG-3′. This amplified a sequence from Exon 15 to Exon 16. 5′- nuclease assays were carried out using Taqman Universal PCR Master Mix from Applied Biosystems according to the manufacturer's instructions and were run on a BioRad Icycler, using the following conditions: 95°C, 10 min,; 40 cycles [95°C, 10 sec; 60°C, 30 sec], 95°C, 1 min.
